# Elevated CO_2_ regulates the Wnt signaling pathway in mammals, *Drosophila melanogaster* and *Caenorhabditis elegans*

**DOI:** 10.1038/s41598-019-54683-0

**Published:** 2019-12-03

**Authors:** Masahiko Shigemura, Emilia Lecuona, Martín Angulo, Laura A. Dada, Melanie B. Edwards, Lynn C. Welch, S. Marina Casalino-Matsuda, Peter H. S. Sporn, István Vadász, Iiro Taneli Helenius, Gustavo A. Nader, Yosef Gruenbaum, Kfir Sharabi, Eoin Cummins, Cormac Taylor, Ankit Bharat, Cara J. Gottardi, Greg J. Beitel, Naftali Kaminski, G. R. Scott Budinger, Sergejs Berdnikovs, Jacob I. Sznajder

**Affiliations:** 1 Division of Pulmonary and Critical Care, Department of Medicine, Feinberg School of Medicine, Northwestern University, Chicago, IL United States of America; 2 Pathophysiology Department, School of Medicine, Universidad de la República, Montevideo, Uruguay; 3 Medical Service, Jesse Brown Veterans Affairs Medical Center, Chicago, IL United States of America; 4 Department of Internal Medicine, Justus Liebig University, Universities of Giessen and Marburg Lung Center, German Center for Lung Research, and The Cardio-Pulmonary Institute, Giessen, Germany; 5 Department of Kinesiology and Huck Institutes of the Life Sciences, The Pennsylvania State University, State College, PA United States of America; 6 Department of Genetics, Institute of Life Sciences, Hebrew University of Jerusalem, Givat Ram, Jerusalem, Israel; 7 Department of Cancer Biology, Dana-Farber Cancer Institute, Boston, MA United States of America; 8 Department of Cell Biology, Harvard Medical School, Boston, MA United States of America; 9 School of Medicine, Systems Biology Ireland and the Conway Institute of Biomolecular and Biomedical Research, University College Dublin, Belfield, Dublin, 4 Ireland; 10 Division of Thoracic Surgery, Department of Medicine, Feinberg School of Medicine, Northwestern University, Chicago, IL USA; 11 Department of Molecular Biosciences, Northwestern University, Evanston, IL United States of America; 12 Department of Internal Medicine, Section of Pulmonary, Critical Care, and Sleep Medicine, Yale School of Medicine, New Haven, CT United States of America; 13 Division of Allergy and Immunology, Feinberg School of Medicine, Northwestern University Feinberg School of Medicine, Chicago, IL United States of America

**Keywords:** Evolutionary genetics, Gene regulation

## Abstract

Carbon dioxide (CO_2_) is sensed by cells and can trigger signals to modify gene expression in different tissues leading to changes in organismal functions. Despite accumulating evidence that several pathways in various organisms are responsive to CO_2_ elevation (hypercapnia), it has yet to be elucidated how hypercapnia activates genes and signaling pathways, or whether they interact, are integrated, or are conserved across species. Here, we performed a large-scale transcriptomic study to explore the interaction/integration/conservation of hypercapnia-induced genomic responses in mammals (mice and humans) as well as invertebrates (*Caenorhabditis elegans* and *Drosophila melanogaster*). We found that hypercapnia activated genes that regulate Wnt signaling in mouse lungs and skeletal muscles *in vivo* and in several cell lines of different tissue origin. Hypercapnia-responsive Wnt pathway homologues were similarly observed in secondary analysis of available transcriptomic datasets of hypercapnia in a human bronchial cell line, *flies* and *nematodes*. Our data suggest the evolutionarily conserved role of high CO_2_ in regulating Wnt pathway genes.

## Introduction

Cells and tissues sense and respond to changes in concentration of gaseous molecules through evolutionarily conserved pathways. Oxygen- and nitric oxide-activated cellular signaling pathways are well described^1,2^, but much less is known about the mechanisms by which non-excitable cells sense and respond to changes in carbon dioxide (CO_2_) concentrations^3,4^. In humans, an increase in CO_2_ levels (hypercapnia) is a consequence of inadequate alveolar gas exchange in patients with lung diseases such as the acute respiratory distress syndrome^5,6^, chronic obstructive pulmonary disease^7^ and others^4,8^. Although initially thought to be benign or even protective^5,6^, it is becoming increasingly evident that hypercapnia has significant pathophysiological effects that may be deleterious to organs such as the lung^7–10^ and skeletal muscles^11^. Recent discoveries suggest that elevation of CO_2_ activates specific signal transduction pathways with adverse consequences for cellular and organismal functions not only in mammals^7,9,12–14^, but also fish^15^, fly *Drosophila melanogaster*^16,17^, and nematode *Caenorhabditis elegans*^17,18^. Hypercapnia has also been reported to alter gene expression in different tissues, cells and species^7,12,16,18,19^. However, a systems-level understanding of elevated CO_2_ effects and of how they are integrated into signaling pathway network, and whether hypercapnia-induced gene programs are similar in different tissues/cells and species is not completely understood.

Here, we performed a large-scale comparative transcriptomic study to explore the interaction/integration/conservation of hypercapnia-responsive genes combining multi-tissue microarray analysis in mice with secondary analysis of transcriptomic datasets of hypercapnia in human bronchial epithelial cells (HBEC)^12^, *Drosophila melanogaster*^16^ and *Caenorhabditis elegans*^18^. We found that hypercapnia activates genes that regulate Wnt signaling in mouse cells, lungs and skeletal muscles. Hypercapnia-activated Wnt pathway homologues were similarly observed in the human bronchial cells, *flies* and *nematodes* at gene expression level. Our data suggest that the role of high CO_2_ as a gaso-signal in regulating Wnt signaling pathways is evolutionarily conserved.

## Results

### Multi-tissue microarray analysis identifies functional similarity in gene networks across different mouse tissues exposed to normoxic hypercapnia

**Figure 1:**
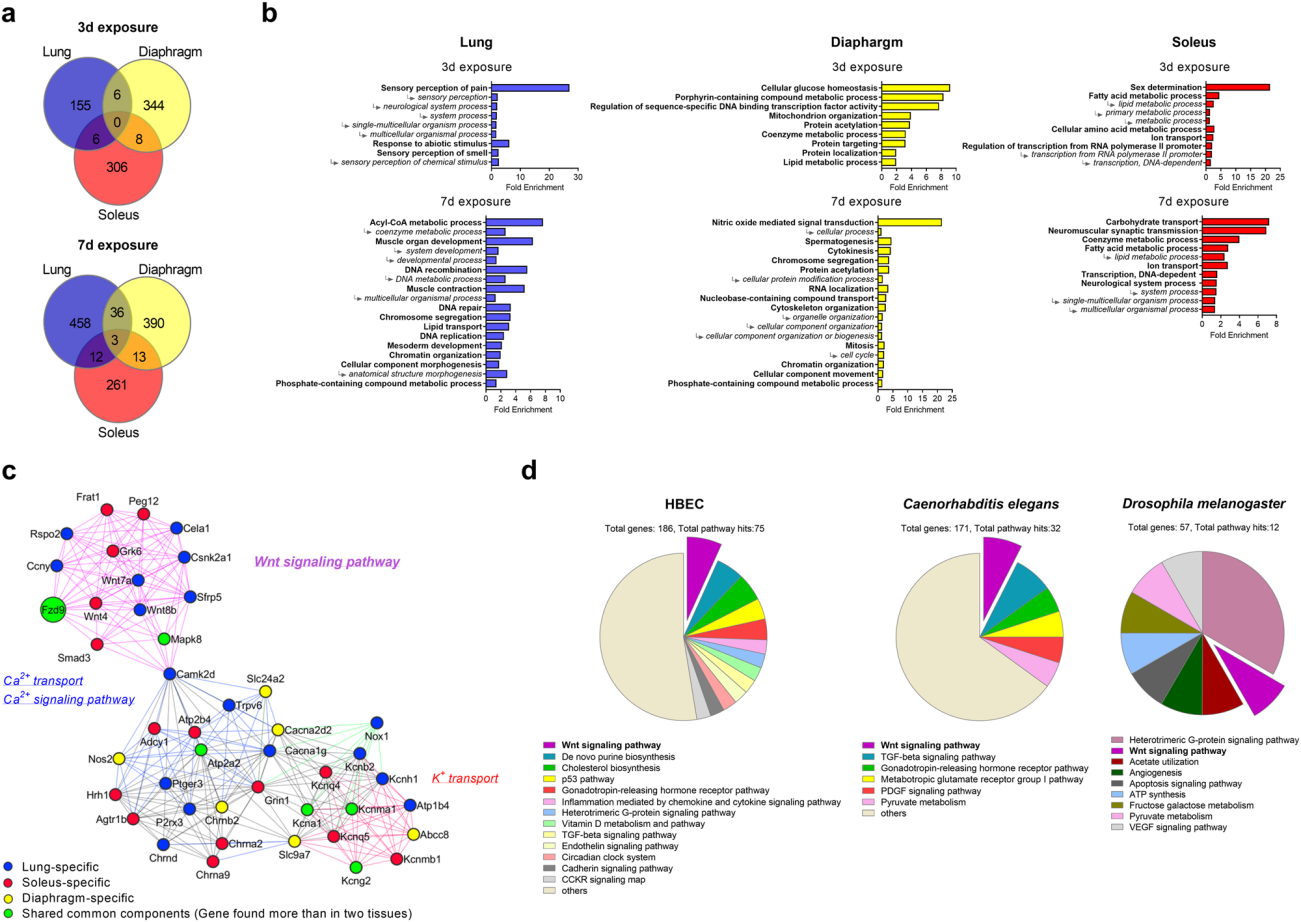
A large-scale transcriptomic study of hypercapnia to combine mouse multi-tissue microarray analysis with secondary analysis of available transcriptomic datasets. (**a–c)** C57BL/6 J mice were exposed to normoxic hypercapnia for 3 (n = 4) or 7 (n = 3) days or maintained in sea-level room air (n = 3). (**a**) Venn diagrams showing the overlap between DEG from lung, diaphragm and soleus. (**b**) Gene ontology analysis of the DEG in each tissue using the PANTHER GO-Slim Biological Process annotation dataset. Arrows indicate hierarchical grouping between GO terms. (**c**) A network diagram constructed from the DEG in each dataset at 7-day exposure conditions. The network diagram revealed groups of genes and pathways that shared common components (green circles) but was also comprised of lung-specific, diaphragm-specific, soleus-specific responsive to hypercapnic exposure (blue, yellow and red circles, respectively). (**d**) Secondary analysis of transcriptomic datasets of hypercapnia in HBEC, *Caenorhabditis elegans* and *Drosophila melanogaster*. PANTHER classification system categorized the DEG in each dataset into signaling pathways.

In mammals, lung diseases are associated with suboptimal function of other metabolic organs including skeletal muscle^20^. To elucidate whether hypercapnia activates conserved genes or gene networks governing specific signaling pathways on an organismal level, we performed a multi-tissue microarray analysis, contrasting available transcriptomic datasets in mouse lungs^7^ with microarray analysis of skeletal muscles, diaphragm and soleus, isolated from mice exposed to normoxic hypercapnia (60 to 80 mmHg = 10% CO_2_ and 21% O_2_) or sea-level room air for up to 7 days (Fig. 1). The microarray analysis in the lungs, as compared to the skeletal muscles, revealed increased number of differentially expressed genes (DEG) dependent on CO_2_ exposure time (Fig. 1a), suggesting that the transcriptional response differs in terms of genes regulated and/or the kinetics of gene activation among the different tissues. Although up-regulated hypercapnia-responsive gene sets differed among the tissues, we found three genes of which was robustly represented in all the tissues, *Fzd9*, *Gm7120* and *LOC100044171*, at 7-day exposure conditions (Fig. 1a and Table S1). We next examined effects of hypercapnia on the functional categorization of the DEG in the tissues. A biological process analysis of the DEG was performed by the Protein ANalysis THrough Evolutionary Relationships (PANTHER) classification system (Fig. 1b). A network diagram constructed from the DEG at 7-day exposure conditions revealed groups of genes and pathways that shared common components (green circles) but was also comprised of lung-specific, diaphragm-specific, soleus-specific response to hypercapnic exposure (Fig. 1c, blue, yellow and red circles, respectively). Despite differences of gene signatures in biological processes observed among the tissues, an unbiased functional analysis of hypercapnia-responsive genes showed that the impact of hypercapnia on gene expression was highly similar. We found three functionally conserved gene networks in response to hypercapnia; Wnt signaling pathway, calcium ion (Ca^2+^) transport/signaling pathway and potassium ion (K^+^) transport (Fig. 1c). To identify transcription factors potentially regulatory for the hypercapnia-responsive genes and conserved in different tissues, we further analyzed our microarray data using an inference transcription factor analysis (Fig. S1). We identified several transcription factors regulatory for hypercapnia-responsive genes, as inferred from differential gene expression signatures of day-3, day-7, or both, hypercapnia responses.

### Analysis of transcriptomic datasets of hypercapnia in a human bronchial cell line and invertebrates

We next compared mouse hypercapnia gene signatures against transcriptomic datasets of normoxic hypercapnia in HBEC^12^, *Caenorhabditis elegans*^18^ and *Drosophila melanogaster*^16^. We need to mention that high CO_2_ exposure conditions in each dataset were somewhat different; 20% CO_2_ exposure for 24 hours in HBEC, 19% CO_2_ for 72 hours in *Caenorhabditis elegans* and 13% CO_2_ for 24 hours in *Drosophila melanogaster*. The PANTHER classification system categorized DEG into signaling pathways in each dataset. Despite different conditions of high CO_2_ exposure in each dataset, we found the DEG involved in Wnt signaling pathway overrepresented in the human bronchial cell line and invertebrates (Fig. 1d). Multiple gene components of the Wnt pathway were also detected in the DEG of mice, HBEC and invertebrates during hypercapnia (Fig. S2 and Table S2). These data suggest that the genes involved in the Wnt pathway are the highest prevalence group of hypercapnia-responsive genes that may be conserved across the tissues and organisms.

### Validation of conserved Wnt pathway genes in the large-scale microarray analysis

**Figure 2:**
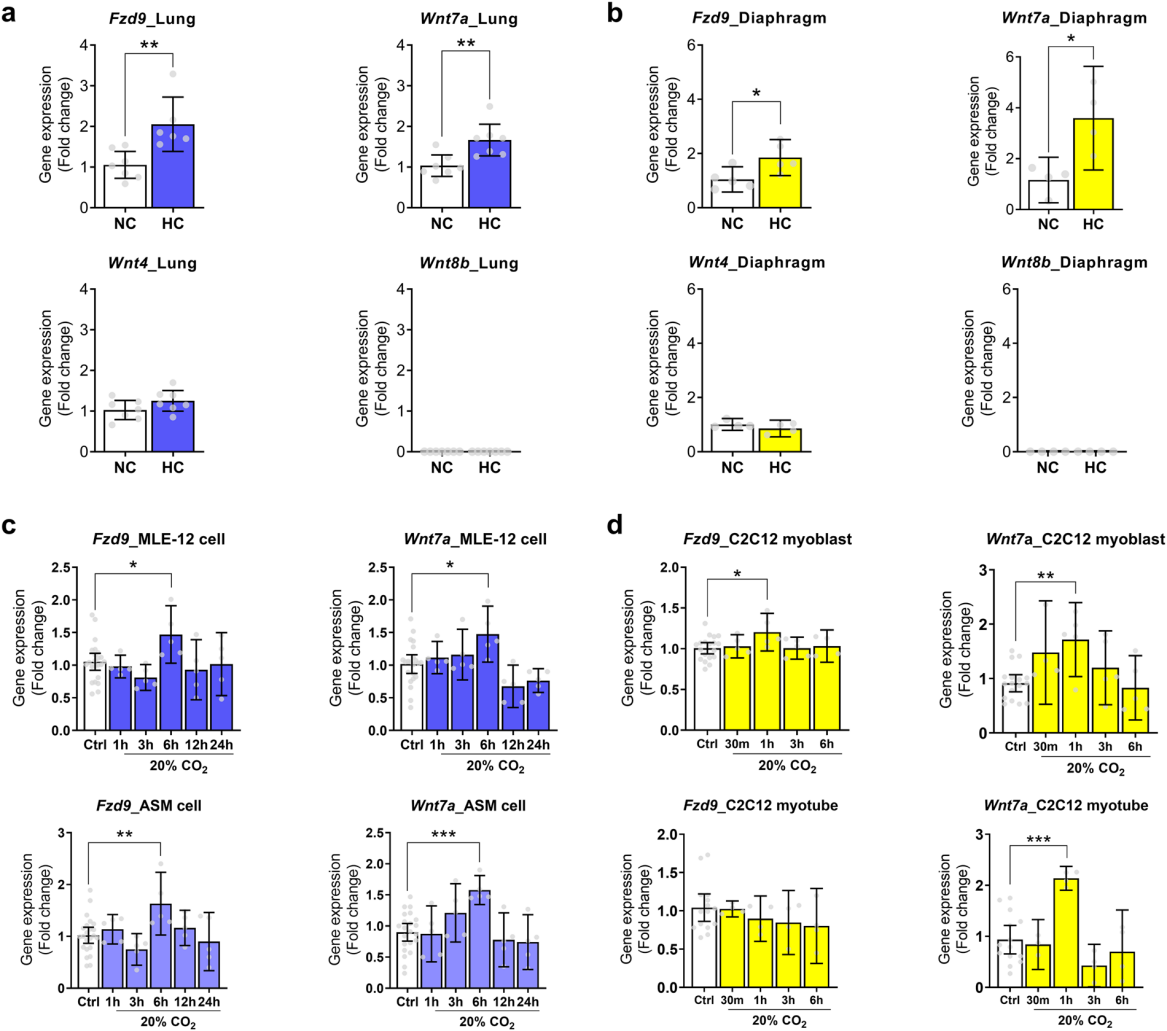
Validation of mouse multi-tissue microarray analysis. (**a,b)** Validation in mouse tissues. *Fzd9*, *Wnt4*, *Wnt7a*, and *Wnt8b* expression in the lung (n = 6–7) (**a**) and diaphragm skeletal muscle (n = 4–5) (**b**) from mice exposed to normoxic hypercapnia for 7 days. NC, normocapnia; HC, hypercapnia. (**c,d)** Validation in mouse lung and skeletal muscle cells. *Fzd9* and *Wnt7a* expressions in MLE-12 cells (Ctrl, n = 22–23; 20%CO_2_, n = 5 per group), ASM cells (Ctrl, n = 22–23; 20%CO_2_, n = 5 per group) (**c**), or C2C12 myoblast (Ctrl, n = 18–19; 20%CO_2_, n = 4–5 per group) or myotube (Ctrl, n = 14–15; 20%CO_2_, n = 3–4 per group) (**d**) exposed to high CO_2_ for up to 24 hours (c) or 6 hours (d). Ctrl, control conditions. All values are represented as mean with error bars shown as the 95% confidence interval. *p < 0.05, **p < 0.01, ***p < 0.001, unpaired two-tailed Student’s t test or one-way ANOVA with Dunnett’s post hoc test.

**Figure 3:**
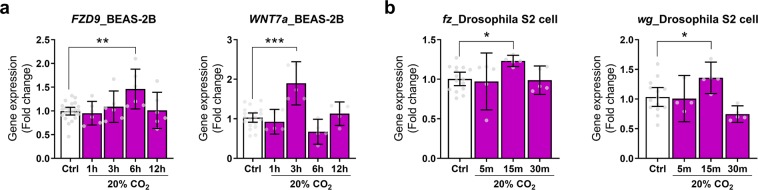
Validation of the transcriptomic datasets of hypercapnia in a human bronchial cell line and invertebrates. (**a)** *FZD9* and *WNT7a* expression in BEAS-2B cells exposed to high CO_2_ for up to 12 hours (Ctrl, n = 20–23; 20%CO_2_, n = 5–6 per group). Ctrl, control conditions. (**b)** *Fz* and *wg* expression in *Drosophila* S2 cells exposed to high CO_2_ for up to 30 min (Ctrl, n = 14–15; 20%CO_2_, n = 4–5 per group). All values are represented as mean with error bars shown as the 95% confidence interval. *p < 0.05, **p < 0.01, ***p < 0.001, one-way ANOVA with Dunnett’s post hoc test.

To validate of our large-scale microarray data, quantitative real-time PCR (qRT-PCR) was performed using RNA isolated from different mouse tissues (lung and diaphragm skeletal muscle) and cells exposed to high CO_2_ conditions (Fig. 2). For *in vivo* experiments, we examined expressions of *Fzd9*, *Wnt4*, *Wnt7a* and *Wnt8b* for the gene network of “Wnt signaling pathway” (Fig. 2a,b). The relative expression levels of *Fzd9* and *Wnt7a* exhibited the same regulatory trends as compared with the microarray analysis, suggesting that hypercapnia activates genes that regulate the Wnt signaling in mice tissues. We next examined expressions of genes validated in the tissues, *Fzd9* and *Wnt7a*, in a panel of mouse cell types; mouse lung epithelial (MLE)-12 cells representing as an epithelial lineage, airway smooth muscle (ASM) cells as the smooth muscle cell lineage and C2C12 myoblasts and myotubes as skeletal muscle lineage exposed to high (~120 mmHg = 20%) CO_2_ and normoxia with an extracellular pH of 7.4. These cell lineages are one of the major cell components of the lung or skeletal muscle tissues and have been reported to show signal transduction pathways and the related-biological effects of hypercapnia as previously reported^7,9,11^. *Fzd9* and *Wnt7a* expressions were tightly regulated and peaked at six hours after high CO_2_ exposure in the lung cells, and at one hour in the skeletal muscle cells but not in the myotubes (Fig. 2c,d). We also examined expression of *FZD9* and *WNT7a* in an immortalized human bronchial epithelial cell line BEAS-2B (Fig. 3a) and major Frizzled and Wnt ligand genes (*fz* and *wg*) in *Drosophila* S2 cells (macrophage like lineage) (Fig. 3b). Consistent with the data in mouse cells, hypercapnia caused transient increases in particular in Frizzled and WNT ligand gene expressions in the human and *fly* cells. Taken together, our data suggest that normoxic hypercapnia activates genes that regulate the Wnt signaling pathway across different cells, tissues and species.

## Discussion

Elevation of CO_2_ has been proposed to affect organismal pathobiology. In recent years, there is accumulating evidence on significant deleterious effects of elevated CO_2_ *per se* on cell, tissue and organismal functions^7,9,11–18,21,22^. However, little is known about the variation in the global transcriptional response to CO_2_ elevation among different cell types, tissues or species. Here, we provide a new systems-level understanding of high CO_2_-conserved effects across *nematodes*, *flies*, mice and humans regulating Wnt signaling pathway genes, which appears to be central to high CO_2_ gaso-signal.

Our data suggest that hypercapnia leads to changes in the expression of genes involved in a variety of biological processes in mouse tissues. Interestingly, the gene network diagram constructed from the hypercapnia-responsive genes among the tissues revealed a functionally similar group of genes that activate Wnt signaling pathway, which was not previously known to be regulated by high CO_2_. The Wnt pathway is a highly conserved signal transduction cascade in animals that has a critical role in many biological processes^23–26^. Wnt signals are also known to activate more than one type of signaling cascade or cross-talk with other signaling pathways, and result in integrated, context-dependent cellular responses^23^. We also observed hypercapnia-responsive Wnt pathway genes that were categorized into other signaling pathways (Table S2). The Wnt signaling pathway may cross-talk with various biological networks as an upstream regulatory signal in response to hypercapnia, which could help explain the significant effects of high CO_2_ on different cells and organisms^3,7,9–18^.

Alterations in expression of Wnt pathway genes may be of central importance in the systems-level understanding of organismal effects and pathobiology of hypercapnia. Despite different exposure conditions of hypercapnia in each microarray dataset, we observed multiple gene components of the Wnt pathway including Inositol trisphosphate receptor gated Ca^2+^ channel and cAMP response element binding protein (CREB) binding protein across species (Fig. S1). Furthermore, we found the hypercapnia-responsive transcription factors such as c-Myc, Oct-4 and c-Jun which are the target genes of the Wnt signaling across mouse tissues exposed to hypercapnia. *In vitro* experiments with the same levels of exposure suggest transient increases in expressions of Frizzled and Wnt ligand genes in cultured cells from different origins including epithelial, smooth muscle, skeletal muscle and macrophage-like lineage in mice, humans and *Drosophila*. Although the magnitude of hypercapnia and gene expression profile of the Wnt signaling pathway differ between each dataset, the biological interpretation of our data point to significant activation of Wnt pathway genes, suggesting an evolutionary role of elevated CO_2_ on Wnt signaling. Wnt signals can activate at least two distinct intracellular signaling, canonical or non-canonical pathways^23^. The canonical Wnt/β-catenin pathway is characterized by cytosolic and nuclear β-catenin accumulation and the activation of certain β-catenin-responsive target genes. The non-canonical β-catenin-independent pathways include the calcium/calmodulin-dependent kinase II (CaMKII)-mediated Wnt/Ca^2+^ pathway and the small GTPase RhoA- and Jun N-terminal kinase (JNK)-dependent planar cell polarity pathway. Specifically, Wnt7a rapidly induces the local activation of CaMKII^26^ and directly interacts with Fzd9 to inhibit cell growth via activation of the JNK pathway^27^. Overexpression of Wnt7a increases expression of the Wnt-target transcription factor genes including c-Myc^28,29^ and c-Jun^28^. c-Myc can bind to the *Fzd9* gene promotor and promotes *Fzd9* expression^30^. Hypercapnia may activate the Wnt7a/Fzd9 signal, setting up a feedback loop via Wnt-target transcription factors that could enhance the Wnt pathway genes. It has also been suggested that Wnt signals activate a metabolic sensor AMP-activated protein kinase (AMPK) in myotubes^24^ and muscle-specific RING finger protein-1 (MuRF-1) leading to muscle atrophy^31^. Interestingly, we have reported that CaMKII, RhoA, JNK, AMPK and MuRF1 are responsive to hypercapnia in physiological contexts. In mammals, hypercapnia impairs cell proliferation^14^ and alveolar fluid reabsorption via a Ca^2+^/JNK pathway^4,9,17^, leads to airway constriction via Ca^2+^/RhoA axis signaling^7^, AMPK/MuRF-1-dependent muscle atrophy^11^, and adipogenesis via CREB activation^13^. Hypercapnia is also known to induce Na,K-ATPase endocytosis in *Drosophila melanogaster*^17^ and lower fertility in *Caenorhabditis elegans*^17^ via activation of the JNK pathway. Together with these reports, our data suggest a linkage of Wnt signaling to the pathobiological changes induced by hypercapnia^4,7,9,11,17^.

Why elevated CO_2_ levels activate Wnt signaling in different tissues/cells and species is not completely understood. Wnt signaling pathway is one of the major pathways regulating tissue architecture during development and in homeostasis of adult tissues^32^. In the mammalian lung system, Wnt signal maintains stemness of alveolar type 2 cells and can trigger transdifferentiation into alveolar type 1 cells which are part of the gas exchange surface of the lung alveolus^33^. We reason that Wnt response during CO_2_ elevation (which occurs in human lung diseases) may represent an adaptive homeostatic mechanism against stress to preserve organismal function during noxious alterations in gaseous (CO_2_) levels. However, such a mechanism may well become maladaptive to cells, organs and organisms as observed in during prolonged hypercapnia^7–18^.

In summary, our transcriptomic analysis of multiple datasets revealed a previously unknown role of hypercapnia in the regulation of gene expression. We found a conserved genomic response to hypercapnia regulating Wnt pathway genes in lung and skeletal tissues and cells in mice, bronchial epithelial cells in humans as well as in *flies* and *nematodes*.

## Methods

### Reagents

All cell culture reagents were purchased from Corning Life Sciences. All chemicals were purchased from Sigma-Aldrich. Reagents for quantitative polymerase chain reaction (qPCR) were purchased from Life Technologies. The mRNA Isolation Kit was purchased from QIAGEN.

### Animals

Adult (9–11 weeks old) C57BL/6J male mice were obtained from the Jackson Laboratories (Bar Harbor, ME). All animals were provided with food and water *ad libitum*, maintained on a 14-hour light/10-hour dark cycle, and handled according to National Institutes of Health guidelines. All of the procedures involving animals were approved by the Northwestern University Institutional Animal Care and Use Committee (IS00000245 and IS00010662). For high CO_2_ exposure, animals were maintained in a Biospherix C-Shuttle Glove Box (BioSpherix) for 3 or 7 days. The chamber’s atmosphere was continuously monitored and adjusted with ProOx/ProCO_2_ controllers (BioSpherix) in order to maintain 10% CO_2_ and 21% O_2_, with a temperature of 20 °C–26 °C and a relative humidity between 30% and 50%. These settings resulted in arterial partial pressure of carbon dioxide (PaCO_2_) of ~80 mmHg and PaO_2_ of ~100 mmHg, whereas in animals maintained in room air PaCO_2_ was ~40 mmHg and PaO_2_ was ~100 mmHg^10,11^. The values of high PaCO_2_ are representative of CO_2_ levels encountered in patients with COPD and mechanically ventilated patients with the “permissive hypercapnia” modality^7,22^. The pH, PaCO_2_, and PaO_2_ values obtained after exposure to 10% CO_2_ for 3 or 7 days were very similar to the values achieved during renal compensation and distinct from acute respiratory acidosis^10,11^. None of the animals developed appreciable distress. At selected time points, animals were euthanized with Euthasol (pentobarbital sodium–phenytoin sodium) and trachea, whole lung, diaphragm and soleus were harvested. Then the tissues were snap-frozen in liquid nitrogen for RNA extraction.

### Mouse multi-tissue microarray

Total RNA from skeletal muscle tissues was isolated with the miRNeasy Mini kit (Qiagen, Valencia, CA). Messenger RNA profiling was performed with an Agilent SurePrint G3 8 × 15 K mouse microarray containing 39,430 messenger RNAs (Sanger miRbase release 9.1), in accordance with the protocol described by the manufacturer (Agilent) and as previously described^34^. Results were compared by unpaired *t* test, and gene expression was considered to be significantly different between groups when *p* < 0.05. For lung transcriptomic analysis, we used the transcriptomic datasets previously described by us^7^. The lists of DEGs in each dataset were obtained by ≥1.4 fold-change with an adjusted p value ≤ 0.05. The identified DEGs were analyzed in the PANTHER classification system (http://www.pantherdb.org/) to determine enriched biological processes and categorize into signaling pathways. Gene signatures representing lung, diaphragm or soleus transcriptome changes in hypercapnia were further subjected to functional gene network analysis. Functional Gene Set Enrichment Analysis (FGSEA) was used to generate functional gene networks by metagrouping of individual gene term sets (referencing GO Biological Process and KEGG Pathways), based on function similarity. The GeneTerm Linker algorithm implemented in the “FGNet” package (R) was used to perform the analysis, which utilized nonredundant reciprocal linkage of genes and biological terms^35^. This methodology filters enrichment output results through reciprocal linkage between genes and terms to produce functional metagroups of key biological significance. Parameters set for this analysis included adjusted p-value < 0.05, minimum gene term support of 3. Genes were deemed “functional hub” genes if they belonged to more than one functional metagroup, suggesting a central role in regulation of biological processes. Networks generated utilizing this analysis were exported in GLM format for further analysis and visualization, using the “iGraph” package (R). Cytoscape 3.2.1 was used for analysis of edge weight, node connectivity, and betweenness within the networks. Transcription factors were not directly measured in our data but inferred from gene expression signatures based on unbiased predictive analysis of known upstream regulators of differentially expressed genes. This analysis was performed in GeneGO Metacore (Thomson Reuters). Venn diagrams were used to determine conserved representation of inferred transcription factors across different tissues and timed responses in hypercapnia.

### Secondary analysis of available transcriptomic datasets of hypercapnia

Data from three studies investigating the transcriptomic response to hypercapnia in human bronchial epithelial cells (GSE110362), *Caenorhabditis elegans*^18^ and *Drosophila melanogaster* (GSE17444) were obtained. Differential expression analysis of each processed dataset was performed with ≥1.4 fold-change with an adjusted p value ≤ 0.05 in the PANTHER pathways classification system.

### Cells lines and culture

MLE-12 cells (CRL-2110; ATCC) were grown in Dulbecco’s modified Eagle’s medium (DMEM) supplemented with 10% fetal bovine serum (FBS), penicillin (100 U/ml), and streptomycin (100 µg/ml; culture medium).

Mouse ASM cell isolation and culture were performed as described elsewhere^7^. Briefly, the trachea from C57BL/6J mice was removed and transferred into culture medium. Connective tissue and airway epithelium were removed by firmly scraping the luminal surface. The trachea strips were cut into small pieces (~1 mm^3^) and cultured in culture medium at 37 °C in 5% CO_2_. ASM cells begin to migrate out of the fragments after 7 to 10 days. The cells were dissociated with 0.05% trypsin and subcultured in culture medium. Identification of mouse ASM cells was based on the morphology and expression of α-SMA. Mouse ASM cells of passage <6 were used in all the experiments.

C2C12 mouse myoblasts (ATCC, CRL1772) were cultured and differentiated as described elsewhere^11^. In brief, cells were allowed to grow in plates until they reached ∼90–95% confluence, and then culture media was changed to DMEM supplemented with 2% horse serum (differentiation media) for C2C12 myotube experiments. The differentiation media was renewed every 18–24 h, and cells were allowed to differentiate for 3 days.

Immortalized human bronchial epithelial cell line BEAS-2B were obtained from ATCC (CRL-9609) and grown in culture medium.

*Drosophila* S2 cells were obtained from the Dr. Silverman’s laboratory (University of Massachusetts Medical School).

### CO_2_ medium and CO_2_ exposure for mammalian cells

For the different experimental conditions, initial solutions were prepared with DMEM/Ham’s F-12 medium/Tris base/MOPS base (3:1:0.25:0.25) containing 10% FBS or 2% horse serum, 100 U/mL penicillin, and 100 µg/mL streptomycin, as described elsewhere^7,11^. The buffering capacity of the medium was modified by changing its initial pH with Tris and MOPS base to obtain a pH of 7.4 at the various CO_2_ levels (pCO_2_ of 40 or ~120 mm Hg). In our prior work, the maximal effects of hypercapnia on signal transduction pathways was achieved at ~120 mmHg of CO_2_ with short (minute to hour) exposure conditions in lung cells^7,9,17^ and skeletal muscle cells^11^, the subsequent cellular experiments with high CO_2_ were performed under these conditions. Lower CO_2_ levels also activate the signaling pathways and have pathophysiologic effects but with more prolonged exposures. The desired CO_2_ and pH levels were achieved by equilibrating the medium overnight in a humidified chamber (C-Chamber, BioSpherix, Lacona, NY). The atmosphere of the C-Chamber was controlled with a PRO CO_2_ carbon dioxide controller (BioSpherix). In this chamber, cells were exposed to the desired pCO_2_ while maintaining 21% O_2_ balanced with N_2_. Before CO_2_ exposure, pH, pCO_2_, and pO_2_ levels in the medium were measured using a Stat Profile pHOx blood gas analyzer (Nova Biomedical, Waltham, MA). Experiments began by replacing the culture medium with the CO_2_-equilibrated medium and incubating in the C-Chamber for the desired time.

### Maintenance of *Drosophila* S2 cells and CO_2_ exposure

*Drosophila* S2 cells were grown at room temperature and protected from light in Schneider’s insect medium containing 10% FBS (Valley Biomedical) and 0.2% Penicillin-Streptomycin (GIBCO). For cell attachment, plates were treated with 1 N HCl for 1 hour, washed 3 times with sterile water, 0.5 mg/mL Concanavalin A (Sigma) for 1 hour, washed once with sterile water. S2 cells were plated at a density of 2.0 × 10^6^ cell per well in six-well plates with the medium and allowed to attach for 1 hour. For high CO_2_ treatments, initial solutions were prepared with Schneider’s insect medium/Tris base/MOPS base (4:0.25:0.25) containing 10% FBS, 100 U/mL penicillin, and 100 µg/mL streptomycin. The buffering capacity of the medium was modified by changing its initial pH with Tris and MOPS base to obtain a pH of 7.2 at the CO_2_ level of ~120 mm Hg. The desired CO_2_ and pH levels were achieved by equilibrating the medium overnight in a C-Chamber protected from light and at room temperature. The atmosphere of the C-Chamber was controlled with a PRO CO_2_ carbon dioxide controller. Before CO_2_ exposure, pH, pCO_2_, and pO_2_ levels in the medium were measured using a Stat Profile pHOx blood gas analyzer. Experiments were started by replacing the culture medium with the CO_2_-equilibrated medium and incubating at room temperature and protected from light for the desired time.

### Quantitative reverse transcription PCR

To isolate total RNA from tissues and cells were homogenized directly in 700 µL of lysis/binding buffer provided by the miRNeasy Mini kit (Qiagen). Complementary DNA was synthesized from 1 μg of total RNA using a qScript cDNA Synthesis kit (Quanta Biosciences, Beverly, MA) and mRNA expression level was determined by quantitative PCR (qPCR) using SYBR Green chemistry (Bio-Rad). Relative expression of the transcripts was determined according to the ∆∆C_t_ method using *Rpl19* for mouse, *RPL19* for BEAS-2B or *RpL32* for *Drosophila* S2 cells as reference for normalization.

### Statistical analysis

Statistical methods are described in the figure legends and in the relevant methods descriptions. Sample size (n) values used for statistical analyses are provided in the relevant figures. Exclusion criteria were pre-established. Individual samples may have been excluded on the basis of sample processing error during experimental work-flow. Statistical outliers were detected and removed based on Grubbs’ test criteria when appropriate. For qRT-PCR data analysis, normally distributed data were analyzed by parametric tests including an unpaired two-tailed Student’s t test for two-group comparisons or a one-way ANOVA for multiple comparisons with Dunnett’s post-hoc corrections for three or more groups. Variances were examined by F test or the Brown-Forsythe test. Statistical analysis was performed using GraphPad Prism (version 7.02, GraphPad Software). p values of < 0.05 were considered to be significant. All values are represented as means with error bars shown as the 95% confidence interval.

## Supplementary information

Figure S1 and Figure S2

Table S1: List of DEG commonly observed in more than two tissues in mice exposed to normoxic hypercapnia for 3 or 7 days.

Table S2: List of hypercapnia-responsive Wnt pathway genes in mice tissues, a human bronchial cell line, Caenorhabditis elegans and Drosophila melanogaster.

Dataset S1: Processed data from mRNA microarray analysis of skeletal muscles isolated from C57BL/6J mice exposed to normoxic hypercapnia for 3 or 7 days.

## Data Availability

The microarray data of mouse skeletal muscle tissues generated in this project can be found in Supplement (Data S1).
