# Supplementary material for: Elevated CO2 regulates the Wnt signaling pathway in mammals, Drosophila melanogaster and Caenorhabditis elegans
Source: Sci Rep. 2019 Dec 3;9:18251. doi: 10.1038/s41598-019-54683-0 (PMC6890671; doi:10.1038/s41598-019-54683-0)
Supplement: Supplementary file 1 — Figure S1 and Figure S2 [file 41598_2019_54683_MOESM1_ESM.docx]

**Elevated CO_2_ regulates the Wnt signaling pathway in mammals, *Drosophila melanogaster* and *Caenorhabditis elegans***

Masahiko Shigemura^1^, Emilia Lecuona^1^, Martín Angulo^2^, Laura A. Dada^1^, Melanie B. Edwards^1^, Lynn C. Welch^1^, S. Marina Casalino-Matsuda^1^, Peter H.S. Sporn^1,3^, István Vadász^4^, Iiro Taneli Helenius^1,10^, Gustavo A. Nader^5^, Yosef Gruenbaum^6^, Kfir Sharabi^7^, Eoin Cummins^8^, Cormac Taylor^8^, Ankit Bharat^9^, Cara J. Gottardi^1^, Greg J. Beitel^10^, Naftali Kaminski^11^, G.R. Scott Budinger^1^, Sergejs Berdnikovs^12^, and Jacob I. Sznajder^1*^

**Affiliations:**

^1^ Division of Pulmonary and Critical Care, Department of Medicine, Feinberg School of Medicine, Northwestern University, Chicago, IL

^2^ Pathophysiology Department, School of Medicine, Universidad de la República, Montevideo, Uruguay

^3^ Medical Service, Jesse Brown Veterans Affairs Medical Center, Chicago, IL

^4^ Department of Internal Medicine, Justus Liebig University, Universities of Giessen and Marburg Lung Center, German Center for Lung Research, and The Cardio-Pulmonary Institute, Giessen, Germany

^5^ Department of Kinesiology and Huck Institutes of the Life Sciences, The Pennsylvania State University, State College, PA

^6^ Department of Genetics, Institute of Life Sciences, Hebrew University of Jerusalem, Givat Ram, Jerusalem, Israel

^7^ Department of Cancer Biology, Dana-Farber Cancer Institute, Boston, MA. Department of Cell Biology, Harvard Medical School, Boston, MA.

^8^ School of Medicine, Systems Biology Ireland and the Conway Institute of Biomolecular and Biomedical Research, University College Dublin, Belfield, Dublin 4, Ireland

^9^ Division of Thoracic Surgery, Department of Medicine, Feinberg School of Medicine, Northwestern University, Chicago, IL

^10^ Department of Molecular Biosciences, Northwestern University, Evanston, IL

^11^ Department of Internal Medicine, Section of Pulmonary, Critical Care, and Sleep Medicine, Yale School of Medicine, New Haven, CT

^12^ Division of Allergy and Immunology, Feinberg School of Medicine, Northwestern University Feinberg School of Medicine, Chicago, IL

**Correspondence:**

Jacob I. Sznajder, M.D.

Division of Pulmonary and Critical Care,

Department of Medicine, Feinberg School of Medicine,

Northwestern University, Chicago, IL, USA

Phone: (312) 908-7737

Fax: (312) 908-4650

Email: [j-sznajder@northwestern.edu](mailto:j-sznajder@northwestern.edu)

**Supplementary figures**

**Figure S1.** Hypercapnia-responsive transcription factors conserved in mouse tissues. Twelve or twenty hypercapnia-responsive transcription factors were observed at 3 day- or 7 day-exposure conditions across mouse tissues, respectively. Eight conserved transcription factors were consistently inferred gene expression signatures in mouse lung, soleus and diaphragm during hypercapnia. TFs, Transcription factors. Red indicates target genes of Wnt signaling.

**Figure S2. Hypercapnia-responsive gene component of WNT signaling pathway in mouse tissues, a human bronchial cell line, *Caenorhabditis elegans* and *Drosophila melanogaster.***

PANTHER classification system categorized the DEG in each dataset into gene component of WNT signaling pathways. The numbers indicate the number of genes in each component.
